# Effect of magnesium oxide on the activity of standard anti-epileptic drugs against experimental seizures in rats

**DOI:** 10.4103/0253-7613.59926

**Published:** 2009-12

**Authors:** Priti Pravin Dhande, Rajani Shrikant Ranade, Balasaheb B. Ghongane

**Affiliations:** Department of Pharmacology, Bharati Vidyapeeth Medical College, Pune, India; 1 Department of Pharmacology, B.J. Medical College, Pune, India

**Keywords:** Epilepsy, magnesium, protective

## Abstract

**Objectives:**

To study the effect of oral magnesium oxide supplementation alone and on the activity of standard anti-epileptic drugs in the animal models of maximal electroshock seizures (MES) and chemically (pentylenetetrazole [PTZ])-induced seizures.

**Methods:**

Healthy male albino rats were given magnesium oxide (MgO) supplementation orally in various doses (500, 750 and 1000 mg/kg /day) for 4 weeks (day 1 to day 28). On day 0 and day 29, response to MES (180 mA for 0.2 s) was tested 1 h after pre-administration of phenytoin or carbamazepine orally. Similarly, in the other groups, the response to PTZ 40 mg/kg i.p. was tested 1 h after pre-administration of oral sodium valproate.

**Results:**

Oral administration of MgO in a low dose (500 mg/kg) for 4 weeks in healthy rats appears to exert protective effect against MES. High oral doses of MgO (750 and 1000 mg/kg) appear to enhance the activity of phenytoin and carbamazepine in the MES model. MgO supplementation was seen to decrease the latency of PTZ-induced seizures.

**Conclusion:**

The dose of oral MgO appears to have an inverse relation with the protective effect in MES-induced seizure model. High doses of MgO supplementation given orally appear to enhance the activity of standard anti-epileptic drugs in the MES-induced seizure model.

## Introduction

Presently, treatment of epilepsy with standard anti-epileptic drugs is associated with a number of shortcomings, like refractoriness in 20–25% patients, dose- and duration-related toxicity and risk of drug interactions with concomitant administration of other drugs.[1,2]

These limitations invite us to study newer agents that would overcome these problems or search for the drugs or substances, which would enhance the efficacy or reduce the dose or toxicity of these standard anti-epileptic drugs.

Anti-epileptic drugs may lead to the depletion of some vitamins and minerals more quickly, thus affecting seizure control, growth or activity. A lowering of cerebrospinal fluid (CSF) or brain magnesium can induce epileptiform activity[3] and there is an association between decreased CSF magnesium and the development of seizures. Significant fall in serum magnesium levels has also been reported in cases of idiopathic epilepsy. The fall is maximum in status epilepticus and severe epilepsy than in mild and moderate epilepsy.[4]

Magnesium has been shown to have a central anticonvulsant effect by blocking N-methyl-D-aspartate (NMDA) receptors in neurons.[5,6] This property is useful in the prevention and management of seizures in pre-eclampsia and eclampsia and control of seizures in epilepsy, hypothyroidism and glomerulonephritis. Magnesium ions and various magnesium salts have been used by different routes like subcutaneous, intraperitoneal, intravenous, intracranial etc., and have shown to exert a significant anticonvulsant effect in experimental models and *in vitro* studies in animals.[7–10] There are a few studies that have shown that combined use of conventional anti-epileptics with NMDA-receptor blockade may have a synergistic effect.[11,12]

The important regulatory role of Mg^2+^ in the central nervous system needs further investigation to evaluate the potential therapeutic advantages of magnesium supplementation in epilepsy.[7,9] It has been shown that magnesium is actively transported across the blood brain barrier[3] and, even on giving magnesium oxide orally, significant serum magnesium levels can be achieved.[7]

Therefore, this study was planned to study the effect of oral magnesium oxide supplementation on the anti-epileptic activity of standard anti-epileptic drugs in rats.

## Materials and Methods

### Animals

Healthy, male, adult albino rats weighing 180–230 g were procured from the Central Animal House of B. J. Medical College, Pune. The general as well as the central nervous system screening of 250 animals was carried out. Finally, 183 rats were included in the study: 120 rats for the study using the maximal electroshock seizures (MES) model and 63 rats for the pentylenetetrazole (PTZ)-induced seizure model, because they responded positively to the respective stimuli during the screening procedure.

### Groups

MES-induced seizure: The various study groups were:

Group I served as control and received only magnesium oxide; Group II and Group III were given phenytoin sodium in high and low dose, i.e. 500mg/kg and 380mg/kg, respectively. Group IV and Group V received carbamazepine in high and low dose, i.e. 50 mg/kg and 35 mg/kg, respectively.

B. PTZ-induced seizure: The various study groups were:

Group VI served as control and received only magnesium oxide; Group VII and Group VIII received sodium valproate in high and low dose, i.e. 1200 mg/kg and 900 mg/kg, respectively.

Each of the above groups had three subgroups, depending on the dose of magnesium oxide used for supplementation orally from day 1 to day 28, i.e. 500 mg/kg/day or 750 mg/kg/day or 1000 mg/kg/day.

### Chemicals

Magnesium oxide (MgO) powder, phenytoin (Dilantin) oral suspension (Parke – Davis, Mumbai, India), carbamazepine (Tegrital) oral suspension (Novartis India Ltd., Mumbai, India) and sodium valproate (Valparin) oral solution (Sanofi Torrent India Ltd., Bangalore, India) were used for the study.

The study was approved by the Animal Ethics Committee, B. J. Medical College, Pune.

### MES-induced seizures

A stimulus of 180 mA for 0.2 s duration was given using the electro-convulsiometer and the responses were tested 1 h after administration of phenytoin or carbamazepine orally.

Parameters observed were time for onset of tonic hindlimb extention (THE), duration of THE, duration of clonic phase and total duration of convulsions.

### PTZ-induced seizures

In the PTZ-induced seizure model, response to PTZ 40 mg/kg i.p. was tested 1 h after administration of sodium valproate orally. The anti-epileptic effect was evaluated by the presence or absence of clonic seizures, time required for onset of seizures and total duration of seizure episode.

### Statistical analysis

The data were analyzed with the SPSS 10.0 (Statistical Package for Social Sciences) software package. The presence or absence of THE response in MES seizures was analyzed by non-Parametric test for two related samples; McNemar test and two-tailed *0*-values were obtained. The paired Students *t*-test was used to compare the data obtained at baseline (day 0) and on day 29 in all the groups for the other parameters noted during the study. A *P*-value of ≤0.05 was considered significant. A linear regression analysis was carried out to check the significance of trend, if observed, in any group.

## Results

### MES-induced seizures

**Figure 1:**
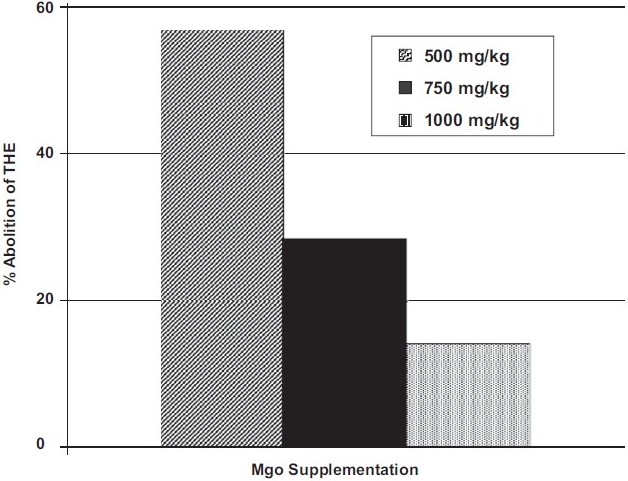
Abolition of tonic hindlimb extension (day 29) after various doses of MgO supplementation in the control group

In the control group, the animals treated with low dose (500 mg/kg) of MgO showed abolition of THE in 57.1%, along with significant reduction in the duration of THE. The total duration of convulsions was also significantly reduced. It appears that the percentage of abolition of THE is inversely proportional to the dose of magnesium oxide used in the present study [Figure 1]. However, this negative linear trend is not statistically significant (r = -0.9820, *P* = 0.121). With the oral supplementation of moderate (750 mg/kg) and high (1000 mg/kg) doses of MgO, there was a significant reduction in the duration of clonic phase and total convulsions [Table 1].

**Table 1:** Responses to MES in rats given magnesium oxide alone (MES method)

*Parameter*		*MgO (500 mg/kg) (n = 7)*	*MgO (750 mg/kg) (n = 7)*	*MgO (1000 mg/kg) (n = 7)*

*Abolition of THE (%)*	*Day 0*	*0*	*0*	*0*
	Day 29	57.1	28.6	14.3
Duration of THE phase (s)	Day 0	11.00 ± 1.62	7.14 ± 1.47	10.29 ± 0.47
	Day 29	4.43 ± 2.10**	7.14 ± 2.18	9.71 ± 1.67
Duration of clonic phase (s)	Day 0	21.43 ± 4.72	26.00 ± 3.01	27.00 ± 2.18
	Day 29	11.71 ± 3.56	11.43 ± 2.41**	10.71 ± 3.01***
Total duration of convulsions (s)	Day 0	32.42 ± 4.98	33.14 ± 3.91	37.28 ± 2.38
	Day 29	16.14 ± 3.83**	18.57 ± 3.29*	20.42 ± 3.21***

Day 0 vs. day 29

* *P* ≤0.05

** *P* ≤0.01

*** *P* ≤0.001, Values indicate mean ± SEM, MgO supplementation given from day 1 to day 28, THE, tonic hindlimb extension

In the phenytoin high-dose group, the animals given moderate and high doses of MgO supplementation showed an increase in the percentage of the abolition of THE [Table 2], which is not significant. However; the duration of clonic phase and total convulsions was significantly reduced. Similar findings were seen in the carbamazepine high-dose group also where, in the rats supplemented with moderate and high doses of MgO, the duration of clonic phase and total convulsions was significantly reduced [Table 3].

**Table 2:** Effect of magnesium on high-dose phenytoin-induced protection (MES method)

*Parameter (post-phenytoin)*		*MgO (500 mg/kg) (n = 7)*	*MgO (750 mg/kg) (n = 7)*	*MgO (1000 mg/kg) (n = 7)*

*Abolition of THE (%)*	*Day 0*	*100*	*85.7*	*71.4*
	Day 29	71.4	100	100
Duration of THE phase (s)	Day 0	0	1.00 ± 1.00	2.00 ± 1.29
	Day 29	2.43 ± 1.60	0	0
Duration of clonic phase (s)	Day 0	27.14 ± 3.62	21.43 ± 2.14	18.71 ± 1.55
	Day 29	27.71 ± 4.17	18.00 ± 1.35*	15.71 ± 1.67*
Total duration of convulsions (s)	Day 0	27.14 ± 3.62	22.42 ± 2.90	20.71 ± 1.39
	Day 29	30.14 ± 3.95	18.00 ± 1.34*	15.71 ± 1.67*

Day 0 vs. day 29

* *P* ≤0.05; values indicate mean ± SEM, MgO supplementation given from day 1 to day 28, THE, tonic hindlimb extension

**Table 3:** Effect of magnesium on high-dose carbamazepine-induced protection (MES method)

*Parameter (post-phenytoin)*		*MgO (500 mg/kg) (n = 7)*	*MgO (750 mg/kg) (n = 7)*	*MgO (1000 mg/kg) (n = 7)*

*Parameter (post-carbamazepine)*	*MgO (500 mg/kg) (n = 8)*	*MgO (750 mg/kg) (n = 8)*	*MgO (1000 mg/kg) (n = 7)*	*71.4*
Abolition of THE (%)	Day 0	100	100	100
	Day 29	75.0	87.5	85.7
Duration of THE phase (s)	Day 0	0	0	0
	Day 29	1.13 ± 0.79	0.38 ± 0.38	0.29 ± 0.29
Duration of clonic phase (s)	Day 0	22.75 ± 3.99	26.38 ± 2.49	27.57 ± 3.04
	Day 29	20.38 ± 2.42	16.88 ± 2.94**	16.57 ± 2.37*
Total duration of convulsions (s)	Day 0	22.75 ± 3.98	28.37 ± 2.48	27.57 ± 3.03
	Day 29	21.5 ± 2.74	17.25 ± 2.74**	16.85 ± 2.25*

Day 0 vs. day 29

* *P* ≤0.05

** *P* ≤0.01; values indicate mean ± SEM, MgO supplementation given from day 1 to day 28, THE, tonic hindlimb extension

**Figure 2:**
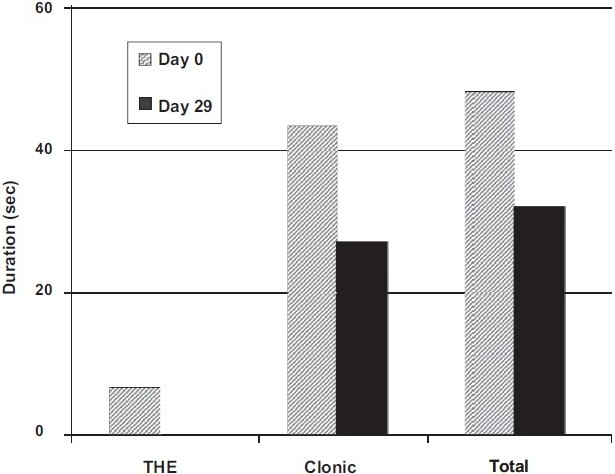
Changes in responses to maximal electroshock seizures after phenytoin low-dose administration in rats given MgO 1000 mg/kg supplementation

The changes in response to MES after phenytoin low dose again showed a significant reduction in the duration of clonic phase and total convulsions with all doses of MgO supplementation [Figure 2], whereas in the carbamazepine low-dose group, these significant results were only seen in the animals given low-dose MgO supplementation.

### PTZ-induced seizures

In the PTZ-induced seizure model, no significant change was observed in any of the parameters in the animals with any of the three doses of MgO. One important finding that was noted was that the onset of seizures was early in all the groups, which was statistically insignificant. [Table 4].

**Table 4:** Effect of magnesium on sodium valproate (low dose)-induced protection (PTZ-induced seizures)

*Parameter (post-valproate)*		*MgO (500 mg/kg) (n = 6)*	*MgO (750 mg/kg) (n = 6)*	*MgO (1000 mg/kg) (n = 6)*
Abolition of clonic convulsions (%)	Day 0	16.6	50	0
	Day 29	0	50	0
Onset of convulsions (s)	Day 0	65.83 ± 15.3	45.5 ± 22.05	69.17 ± 12.74
	Day 29	55.5 ± 2.4	29.5 ± 14.91	39.17 ± 3.00
Duration of seizure (s)	Day 0	88.33 ± 19.69	50.83 ± 26.66	80.00 ± 25.43
	Day 29	79.17 ± 9.12	35 ± 18.75	75.00 ± 11.18

Day 0 vs. day 29: not significant; values indicate mean ± SEM, MgO supplementation given from day 1 to day 28

## Discussion

The present study demonstrates that oral administration of magnesium oxide (MgO) for 4 weeks in healthy rats appears to exert a protective effect against MES, which appears to have an inverse linear relation with the doses used. These results suggest that supplementation with MgO will have a seizure protective role: the dose used being critical, and needs to be decided.

Many studies have shown the anticonvulsant effect of Mg^2+^ using different experimental and *in vitro* models. Magnesium helps in slowing the spread of electrical discharge from one area of the brain to the rest and therefore magnesium depletion can cause a marked irritability of the nervous system, eventually resulting in epileptic seizures.[3,4] The magnesium depletion here refers to that in the CSF or in the brain, which is associated with the development of seizures.[3,13] CSF magnesium concentrations can readily be repleted following magnesium supplementation,[3] and this may explain the protective effect observed in the control groups after a 4-week supplementation with oral MgO.

There has been a recent resurgence of interest in magnesium deficiency and its neurological consequences due to the finding that magnesium, at physiological concentrations, blocks NMDA receptors in the neurons.[14] The NMDA receptor is the most well-characterized excitatory amino acid receptor subtype. The NMDA receptor channel complex consists of four components, one of which is a cation-binding site located inside the channel, which is occupied by Mg^2+^ at physiological concentrations. The normal function of the NMDA receptor complex depends on the dynamic equilibrium among its various parts. Loss of equilibrium may block the function of the entire system and result in the expression of excitotoxicity.[13]

Some authors[15] suggest that Mg^2+^ deficiency, although not necessarily responsible for the convulsive activity, may contribute to facilitating an epileptic episode or may lead to more severe convulsions.

One proposed mechanism for magnesium's suppressive action on the NMDA receptor is that Mg^2+^ enters the channel and blocks the passage of more permeable ions, such as Ca^2+^, in a voltage-dependent manner.[5,14] A second possibility of magnesium's central anticonvulsant effect is that Mg^2+^ competes with Ca^2+^ uptake pre-synaptically, reducing calcium-dependent neurotransmitter release.[13] This is also supported by the fact that magnesium is a physiological calcium blocker.[16]

From this study, it appears that the higher doses of MgO supplementation enhance the activity of phenytoin and carbamazepine in the MES seizure model. In fact, the low and high doses of phenytoin and carbamazepine were used in the present study in order to gather more information regarding the exact role of magnesium. The oral route was used in the current study because it is the route for long-term therapy. However, it is now evident that these drugs have to be used parenterally in order to delineate their interaction with MgO in future studies.

Two studies performed using competitive NMDA-receptor antagonists concluded that NMDA-receptor blockade leads to the enhanced anticonvulsive action of conventional anti-epileptics against MES-induced seizures.[11,17] Another study[12] indicates that glutamate receptor (NMDA) antagonists differentially affect the protective activity of conventional anti-epileptics (carbamazepine, phenobarbital, diphenylhydantoin and valproate) against amygdala-kindled seizures in rats.

Anti-epileptic drugs used to control seizures often lead to the depletion of certain minerals (including Mg^2+^).[18,19] When magnesium deficiency is the underlying issue, the seizures are usually resistant to medication and, the longer the patient is untreated by magnesium, the more permanent is the damage. Magnesium deficiency can readily be corrected by magnesium supplementation in high doses[3] and the same reason can be applied to the beneficial effects observed in the study.

In the present study, oral magnesium oxide failed to produce any significant change in PTZ-induced seizures with especially moderate and low doses. The MgO supplementation has, in fact, reduced the seizure latency in the animals, especially with the high doses, although not significantly (*P* = 0.06). Some studies[7] have reported that the anticonvulsive effect of Mg^2+^ is not observed with PTZ-induced convulsions. In another study,[20] it has been shown that NMDA antagonists block the tonic but not the clonic component of seizures.

Sodium valproate in high doses was able to protect all the animals against PTZ-induced seizures on day 0. Therefore, this group was not followed as there were no parameters to compare on day 29. The animals supplemented with various doses of magnesium and given sodium valproate at a low dose showed early onset of seizures, although not statistically significant. These observations are parallel to the results seen with the group given magnesium alone.

Thus, it is apparent from the present study that Mg^2+^ (given in the form of MgO) has beneficial effects only in the MES-seizure model and undesired effects in the PTZ-seizure model. Therefore, it is likely that Mg^2+^ plays an important positive role in grand-mal epilepsy rather than the petit-mal epilepsy.

Further studies with lower doses of MgO supplementation (higher range of normal intake of Mg^2+^, i.e. physiological or just supraphysiological doses) along with parenteral administration of drugs effective against the MES-seizure model are suggested to clarify further the role of Mg^2+^ in the management of epilepsy.
